# With a pinch of extra salt—Did predatory protists steal genes from their food?

**DOI:** 10.1371/journal.pbio.2005163

**Published:** 2018-02-02

**Authors:** Laura Czech, Erhard Bremer

**Affiliations:** 1 Department of Biology, Laboratory for Molecular Microbiology, Philipps-University Marburg, Marburg, Germany; 2 LOEWE-Center for Synthetic Microbiology, Philipps-University Marburg, Marburg, Germany

## Abstract

The cellular adjustment of Bacteria and Archaea to high-salinity habitats is well studied and has generally been classified into one of two strategies. These are to accumulate high levels either of ions (the “salt-in” strategy) or of physiologically compliant organic osmolytes, the compatible solutes (the “salt-out” strategy). Halophilic protists are ecophysiological important inhabitants of salt-stressed ecosystems because they are not only very abundant but also represent the majority of eukaryotic lineages in nature. However, their cellular osmostress responses have been largely neglected. Recent reports have now shed new light on this issue using the geographically widely distributed halophilic heterotrophic protists *Halocafeteria seosinensis*, *Pharyngomonas kirbyi*, and *Schmidingerothrix salinarum* as model systems. Different approaches led to the joint conclusion that these unicellular Eukarya use the salt-out strategy to cope successfully with the persistent high salinity in their habitat. They accumulate various compatible solutes, e.g., glycine betaine, myo-inositol, and ectoines. The finding of intron-containing biosynthetic genes for ectoine and hydroxyectoine, their salt stress–responsive transcription in *H*. *seosinensis*, and the production of ectoine and its import by *S*. *salinarum* come as a considerable surprise because ectoines have thus far been considered exclusive prokaryotic compatible solutes. Phylogenetic considerations of the ectoine/hydroxyectoine biosynthetic genes of *H*. *seosinensis* suggest that they have been acquired via lateral gene transfer by these bacterivorous Eukarya from ectoine/hydroxyectoine-producing food bacteria that populate the same habitat.

## Introduction

**Fig 1:**
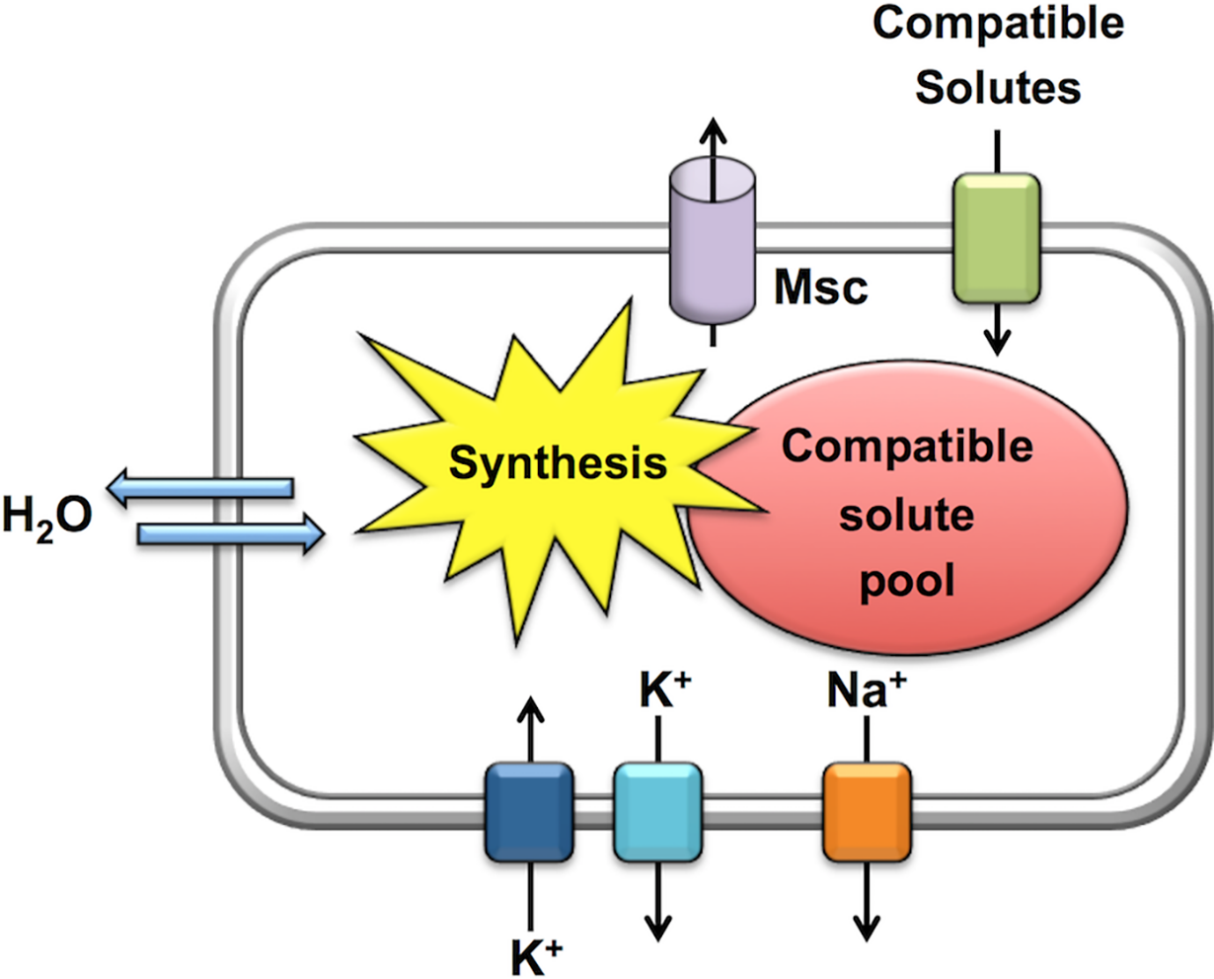
Schematic illustration of the salt-out adjustment strategy of a microbial cell to hyper- and hypoosmotic challenges. Msc, mechanosensitive channels.

The invention of a semipermeable cytoplasmic membrane was a key event in the development of primordial cells because it provided a privileged space for the faithful copying of the genetic material, a reaction vessel for biochemical transformations, and for energy generation to fuel growth. The cytoplasm of microorganisms is a highly crowded compartment caused by large concentrations of nucleic acids, proteins, and metabolites [1,2]. Together, these compounds generate a considerable osmotic potential and thereby instigate osmotically driven water influx, a process that, in turn, causes the buildup of a hydrostatic pressure, the turgor [2–4], in walled microbial cells. Decreases and increases in the external osmolarity are ubiquitous environmental cues and stressors that affect growth and survival of many organisms [5]. Cellular response to fluctuations in external osmolarity have been particularly intensively studied in bacteria (Fig 1) [2,6,7], and these studies also provided a basic framework for an understanding of key osmotic stress adaptation strategies employed by eukaryotic cells [8–11].

Maintenance of turgor is considered critical for the growth of microorganisms [1–4,12], but the semipermeable cytoplasmic membrane makes cells vulnerable to fluctuations in the external osmolarity and salinity, as cells seem to strive to attain crowding homeostasis and thus turgor within physiologically acceptable boundaries [1]. Hyperosmotic conditions result in water efflux, plasmolysis, and reduction in vital turgor [5]. Conversely, hypoosmotic circumstances trigger water influx and thereby cause an undue rise in turgor that can lead in extreme cases to the rupture of the cell [3]. In some organisms, dedicated water channels, the aquaporins, allow accelerated water fluxes across the cytoplasmic membrane in response to external osmotic changes [13], but their potential physiological role in the adaptation of microorganisms to fluctuations in the external osmolarity is not truly understood [14].

Because no cell can actively (e.g., through expenditure of energy) pump water across the cytoplasmic membrane in a directed fashion, adjustment in the proper hydration of the cytoplasm has to rely on indirect measures to direct water fluxes [1–3,5]. To accomplish this under hyperosmotic conditions, microbial cells accumulate ions through transport and synthesize or import physiologically compliant organic osmolytes [15], the compatible solutes (Box 1), to promote water retention and influx (Fig 1) [2,6,7]. Conversely, under hypoosmotic conditions, the cell rapidly expels these compounds through the transient opening of mechanosensitive channels (Msc) (Fig 1) to curb excessive water influx and to prevent a potentially nonsustainable increase in turgor [3,16].

**Box 1. Biological function, molecular mechanism, and diversity of compatible solutes**

Compatible solutes are operationally defined as organic osmolytes that can be amassed to exceedingly high cellular concentrations without disturbing vital biochemical and physiological processes [17]. They are therefore widely used by Bacteria, Archaea, and Eukarya to counteract the high-salinity–and/or high-osmolarity–instigated water efflux from the cytoplasm [6–8,18]. The physicochemical attributes of compatible solutes make them highly compliant with the functionality of macromolecules and cellular components. With respect to proteins, compatible solutes operate against their denatured state and thereby promote proper hydration, folding, and stability [15,19,20], effects that led to their description as chemical chaperones. Studies into the mechanism(s) through which these stabilizing organic osmolytes act revealed that they are strong water structure formers and are preferentially excluded from the immediate hydration shell of the protein backbone [15]. For thermodynamic reasons, the uneven distribution of these solutes in the cell water generates driving forces acting against protein denaturation and aggregation [19, 20] and thereby favours the well-folded functional state of macromolecules [15, 21].

Compatible solutes synthesized by members of the Bacteria are chemically diverse but are all low–molecular weight uncharged or zwitterionic organic compounds and are highly water soluble [6,7,18]. They can be grouped into the following different chemical classes: (i) sugars (e.g., trehalose, sucrose), (ii) polyols (glycerol, myo-inositol, glycosylglycerol), (iii) amino acids and their derivatives (L-proline, L-glutamate, ectoines), (iv) quaternary amines (e.g., glycine betaine, arsenobetaine, L-carnitine, proline-betaine, trimethylammoniumoxide) and their sulfonium analogues (dimethlysulfoniopropionate [DMSP], taurine), (v) sulfate esters (e.g., choline-*O*-sulfate), and (vi) small peptides (*N*-acetyl-glutaminyl-glutamine amide) [22]. Some of these compatible solutes occur rarely in microorganisms, while others are very abundant in nature [5–7,18,22]. An example of the latter group is DMSP, a sulfur-containing compound that is produced in millions of tons in marine habitats by phytoplankton and macroalgae as an osmostress protectant and whose volatile metabolite dimethylsulfide (DMS) is a highly relevant climate gas [23].

Compatible solutes not only serve as effective stress protectants [6–8,18,22] but they are also widely used as nutrients by microorganisms [24]. They are released into the environment as a consequence of sudden osmotic down-shifts through the transient opening of Msc [3]; after the attack and ensuing cell lysis of microorganisms by phages or toxins; through the grazing activities of protozoa on their microbial prey; and through root exudates, decomposing plant material, and urine of mammals [24]. The release of these compounds from the producer cells provides new opportunities for organisms living in the same ecosystem because they can be acquired through high-affinity transport systems either for their reuse as stress protectants [5, 7] or as valuable nutrients [24].

## Msc: Emergency relief valves

The opening and closing of Msc occurs within milliseconds and is a passive cellular response that is triggered by increased tension in the lateral plain of the cytoplasmic membrane upon osmotically instigated water influx [3, 16]. Often, multiple types (Msc mini [MscM], Msc small [MscS], Msc large [MscL]) of these safety valves are found in a given microbial cell. They possess different pore sizes and gating behaviour, thereby providing the cell with a finely graded adjustment response to the severity of the imposed osmotic down-shift [3,16]. Msc are essential under severe osmotic down-shock conditions but not under steady-state growth of microorganisms at high osmolarity [3]. This finding indicates that the stress-bearing peptidoglycan sacculus of gram-positive and gram-negative bacteria [25] is by itself insufficient to restrain the practically instantaneous increase in turgor that occurs during a rapid transition from high to low external osmolarity [3, 16].

## The salt-in and salt-out strategy

To cope with sudden or sustained increases in the environmental osmolarity and/or salinity, microorganisms have developed two strategies that are generally referred to as the salt-in and salt-out response [5–7]. In contrast to the passive release of solutes from osmotically down-shocked cells via the transient opening of Msc, adjustment to high-osmolarity surroundings requires active countermeasures to maintain a properly hydrated cytoplasm and a physiologically adequate level of turgor [1,2,6,7]. A restricted number of Bacteria and Archaea that primarily live in permanently high-saline ecosystems use the salt-in strategy and amass molar concentrations of K^+^ and Cl^−^ ions on a permanent basis to balance the osmotic gradient across their cytoplasmic membrane [26]. While energetically favourable [27], the ensuing high–ionic strength cytoplasm forced adjustments of the entire proteome in order to maintain the biological functionality of all extra- and intracellular components [26,28,29]. On an evolutionary timescale, this has left an acidic signature on the proteome with a narrow distribution of isoelectric points as the consequence of reduced hydrophobicity of proteins and a strong increase in negatively charged amino acids exposed on protein surfaces. In a cytoplasm highly enriched in K^+^/CL^−^ ions, these modifications increase protein hydration, solubility, stability, and functionality and were thought to be coupled with obligate protein halophilicity [26,28,29].

**Fig 2:**
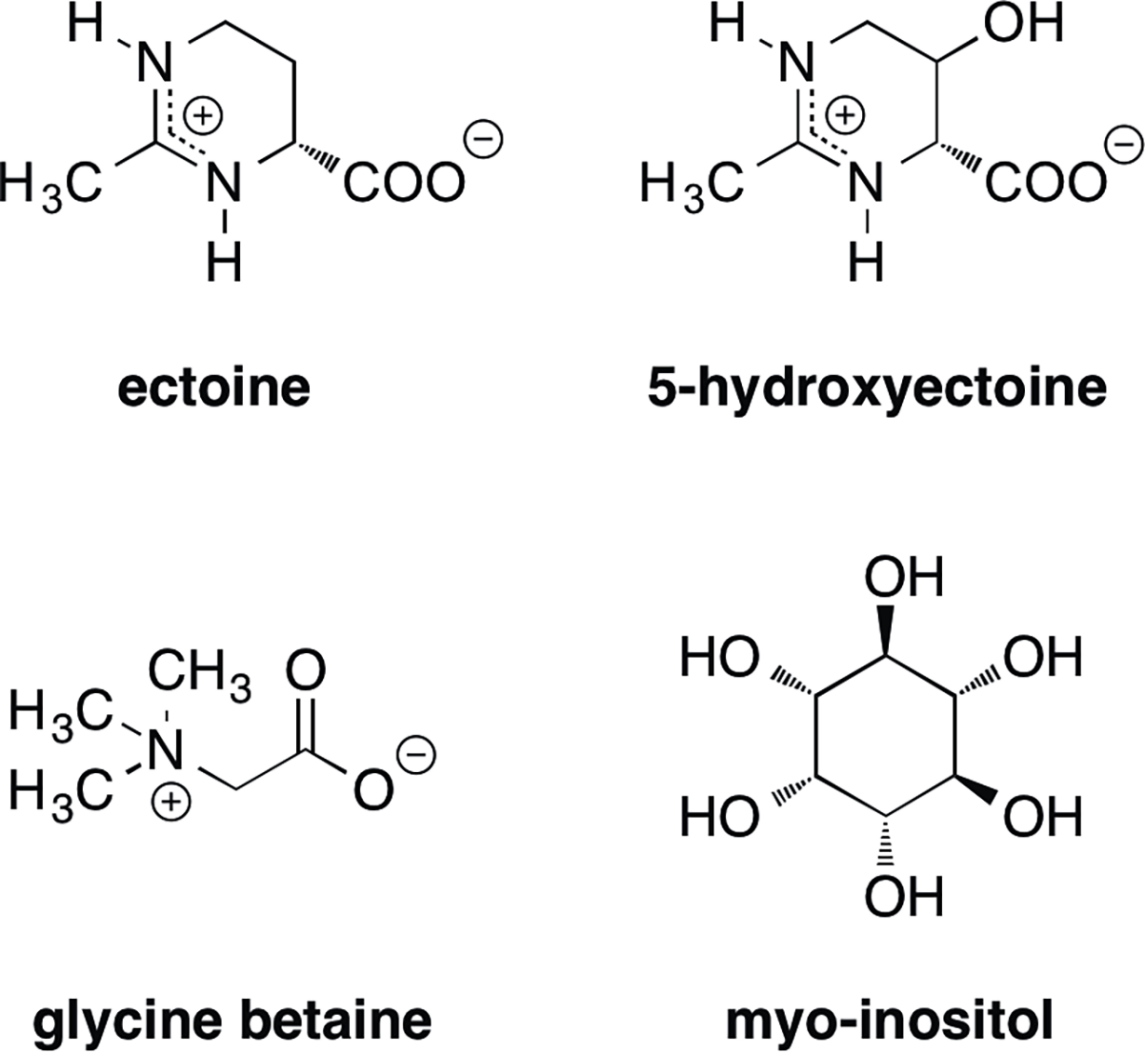
Chemical structures of selected compatible solutes.

The identification of an acidic proteome was considered as predictive for the use of the salt-in osmostress adaptation strategy. While this is probably correct in general, recent findings led to modifications of this long-held view [26] because a considerable number of Halobacteriales, a group of halophilic Archaea, were discovered that combine a high-K^+^ cytoplasm with the accumulation of the compatible solutes glycine betaine (Fig 2) and trehalose/2-sulfotrehalose [30]. Furthermore, a highly acidic proteome of the photosynthetic anaerobic proteobacterium *Halorhodospira halophila*, one of the most halophilic microorganisms known, was observed, and the cytoplasmic K^+^ content of the cells was strongly regulated by the salinity of the growth medium. The K^+^ content of the cells (molar concentration) was very high at high salinity (35%) but rather moderate (mM concentration; at the level of that observed for the non-halophile *Escherichia coli*) at modest external salinities (5%) [31]. Consequently, this finding challenges the traditional view that obligate protein halophilicity is the price evolution has to pay for the salt-in osmostress adaptive strategy and prompts the exploration of new avenues of research into the evolution of microbial cellular adjustment strategies to high-salinity environments and the physiological consequences of a highly acidic proteome [26,31].

Microorganisms that employ the salt-out strategy also import large amounts of K^+^ ions as an initial, rapid osmostress response, but they avoid their long-term accumulation under sustained osmotic stress conditions [5,7]. Export of cytotoxic Na^+^ that can enter the cell through various processes (e.g., via Na^+^-coupled transporters for compatible solutes [5]) is also a very important attribute for maintaining a low–ionic strength cytoplasm (Fig 1) [2,7]. Hence, instead of ions, microbial salt-out adopters balance the osmotic gradient across their cytoplasmic membrane primarily through the high-level accumulation of various types of compatible solutes (Box 1) [2,6,7,15]. This allows them to subsequently reduce the K^+^ pool through export (Fig 1) without compromising the level of the osmotic potential of the cytoplasm required for growth and maintenance of turgor [1,2,4,12]. The accumulation of compatible solutes also counteracts protein misfolding and aggregation that will occur upon the imposition of osmotic stress (Box 1) [15,19,20]. The amassing of compatible solutes can be accomplished either via osmotically stimulated synthesis or through uptake from environmental sources via osmotically controlled transport systems [2,6,7]. Although synthesis and import of compatible solutes is, from an energetic point of view, far more demanding than the salt-in mechanism [26], it provides a considerable degree of flexibility to salt-out adopters because it does not tie them ecophysiologically to permanently high saline and/or osmolarity habitats. Collectively, these microorganisms adapt to both sudden and sustained increases in environmental osmolarity through coordinated changes in their genome-wide transcriptional profile, through the osmostress-responsive induction of genes for compatible solute importers, and modulation of the activity of these transport systems [2,5–7].

## Relief from osmotic stress through synthesis of the compatible solutes glycine betaine and ectoine

The trimethylammonium compound glycine betaine (Fig 2) is probably the most widely used compatible solute on Earth because both Pro- and Eukarya employ it as a highly effective protectant against osmotic and other types of stresses (Box 1) [18]. It can be synthesized under aerobic conditions through the oxidation of choline, with glycine betaine aldehyde as the intermediate. Bacteria, Archaea, Fungi, plants, and animals are able to synthesize it from this precursor, and a combination (or types) of various enzymes can be used. In microorganisms, choline typically needs to be imported because most of them lack the ability to synthesize this molecule de novo. Some Bacteria and Archaea can also produce glycine betaine through the sequential methylation of glycine [32], but this is not the preferred route for synthesis because of the high energy demand to regenerate the cofactor [*S*-adenosylmethionine (SAM)] needed for the activity of the two enzymes that catalyze the methylation of glycine [32]. Both the choline to glycine betaine oxidation pathway and the de novo synthesis route from glycine confer a considerable degree of osmostress resistance to microbial cells [5,7,32].

In contrast to the widely distributed glycine betaine molecule in Pro- and Eukarya, the compatible solute ectoine and its derivative 5-hydroxyectoine (Fig 2) were initially perceived as rather specialized microbial osmostress protectants. However, the discovery of their biosynthetic genes [33] and subsequent extensive database searches revealed their wide distribution in genomes of members of the Bacteria [34]. Lateral gene transfer, a major driver of microbial evolution [35], is probably responsible for the introduction of the *ect* biosynthetic genes into selected members of the archaeal genera *Nitrosopumilus*, *Methanothrix*, and *Methanobacterium* [36].

Synthesis of ectoine proceeds from L-aspartate-beta-semialdehyde and involves the transformation of this central metabolite by the sequential enzyme activities of diaminobutyrate-2-oxoglutarate aminotransferase (EctB; EC 2.6.1.76), diaminobutyrate acetyltransferase (EctA; EC 2.3.1.178), and ectoine synthase (EctC; EC 4.2.1.108) [33]. A subgroup of ectoine producers also synthesize the ectoine derivative 5-hydroxyectoine (Fig 2), an effective compatible solute in its own right that is often endowed in vivo and in vitro with additional or enhanced stress protective properties in direct comparison with ectoine [33, 34]. All 5-hydroxyectoine producers are aerobic, or at least O_2_ tolerant, microorganisms because the ectoine hydroxylase (EctD; EC 1.14.11.55) is an oxygen-dependent enzyme [34, 36]. The ectoine biosynthetic genes (*ectABC*) are typically organized as an osmotically inducible operon [37] that may also contain the gene (*ectD*) for the ectoine hydroxylase and a specialized aspartokinase (Ask_Ect) involved in synthesizing the ectoine precursor L-aspartate-beta-semialdehyde [34,36]. Effective osmostress protection can also be accomplished through the import of ectoines, and several types of osmotically induced ectoine/5-hydroxyectoine import systems have been identified in microorganisms (for an example see [38] and additional references therein).

## Halophilic protists—How do they do it?

Marine and hypersaline habitats are populated not only by a physiological and taxonomically diverse group of Bacteria and Archaea [26, 27], but halophilic protists are also ecophysiologically critical inhabitants of these challenging ecosystems [39–41]. They serve crucial roles as primary producers and decomposers, and some of them exert through their bacterivorous activity a major influence on biological diversity and abundance of bacteria in these habitats [41]. While the cellular adjustments to osmotically challenging environments have been intensively studied over the years in Bacteria and Archaea [2,5–7], a thorough understanding of the cellular osmostress responses of halophilic unicellular eukaryotes lags far behind [41]. However, an in-depth understanding of these organisms is highly desirable because they are not only very abundant in nature but also represent the majority of eukaryotic lineages [39,41,42]. Studies investigating the osmostress adaptation strategies in the green algae *Dunaliella salina* [11], in the baker’s yeast *Saccharomyces cerevisiae* [9], and in some halotolerant/halophilic fungi (e.g., *Hortaea werneckii*, *Wallemia ichthyophaga*) [10] have already been conducted in quite some detail.

Three recent publications now lay the groundwork to unravel the physiology and molecular biology of osmostress adaptation strategies of halophilic heterotrophic protists [41,43–45] and thus allow a comparison with their halophilic or halotolerant bacterial counterparts. These studies focused on *Halocafeteria seosinensis*, *Pharyngomonas kirbyi*, and *Schmidingerothrix salinarum*, phagotrophic protists that were originally isolated on different continents (Europe, Northern America, Asia), but their common habitat are hypersaline solar ponds, habitats subjected to evaporation and thus leading to gradual increases in salinity that can range between seawater and saturated brines [41]. The reports by Harding et al. [43,44] and Weinisch et al. [45] do not only allow a glimpse into the basic features of the osmostress responses used by these species but also hold several surprises for scholars of microbial osmostress adjustment systems.

Studies with *H*. *seosinensis* and *P*. *kirbyi* showed that their cytoplasmic proteomes are not particularly highly acidic but possess a more hydrophilic signature than eukaryotic microbes inhabiting marine environments, thereby suggesting that they possess a somewhat higher steady-state salt content than marine protists [43]. Consistent with the finding from proteomic studies are data derived by Weinisch et al. [45] using an ion imaging approach. These experiments showed that the cytoplasmic K^+^ and Na^+^ content of *S*. *salinarum* do not change significantly in response to increases in the external salinity. The conclusions that the three studied halophilic protists do not use a salt-in approach are also supported by experiments assessing the inhibiting effect of salinity on enzyme activity of the *S*. *salinarum* malate and isocitrate dehydrogenases [45]. However, the occurrence of taxonomically diverse protists capable of growing in habitats of distinct salinities raises the possibility that various lineages of unicellular Eukarya have evolved different adaptation strategies to high-salinity environments [41].

Studies probing the transcriptional response of *H*. *seosinensis* to high-salinity challenges revealed the involvement of various stress responses (e.g., chaperones that repair misfolded proteins), genes for neutralizing the detrimental effects of oxidative stress, K^+^ and Na^+^ transporters critical for ion homeostasis, metabolism and transport of lipids (e.g., sterol biosynthesis), carbohydrate and amino acid metabolism, and signal transduction pathways, including G-protein–coupled receptors [44]. The studies by Harding et al. [43,44] focusing on *H*. *seosinensis* and *P*. *kirbyi* and that of Weinisch et al. [45] on *S*. *salinarum* show that the synthesis and import of compatible solutes (Box 1) make major contributions to their salt stress adaption.

The genome-wide transcriptome analysis of salt-stressed *H*. *seosinensis* cells yielded the totally unexpected finding that predicted biosynthetic genes for the compatible solutes ectoine/5-hydroxyectoine are present in the chromosome of this halophilic protist and that their transcription is up-regulated in response to salt stress [43, 44]. Ectoines (Fig 2) have so far been considered as compatible solutes exclusively synthesized and used by Bacteria and by a few Archaea [33,34,36]. Because *H*. *seosinensis* and *S*. *salinarum* are bacterivorous, the detection of DNA sequences in *H*. *seosinensis* related to microbial genes is at least initially of some concern because their misinterpretation as of eukaryotic origin can seriously compromise assembly into DNA scaffolds and the interpretation of biological findings [46–48]. However, with respect to the data reported by Harding et al. [43] for *H*. *seosinensis*, one can rest easy because each of the ectoine (*ectABC*) and hydroxyectoine (*ectD*) biosynthetic genes and the specialized aspartokinase (*ask_ect*) harbor spliceosomal introns, genetic elements that are not found in Bacteria and Archaea [49]. Because microbial ectoine/5-hydroxyectoine producers frequently populate marine and hypersaline habitats [33,34,36], a reasonable hypothesis for the occurrence of ectoine/5-hydroxyectoine biosynthetic genes in *H*. *seosinensis* is their acquisition via horizontal gene transfer from a prokaryotic donor and their subsequent adaptation to the genomic context and transcriptional profile of a eukaryotic host cell. The detection of predicted N-terminal mitochondrial targeting sequences of the ectoine/5-hydroxectoine biosynthetic enzymes from *H*. *seosinensis*, all of which are cytoplasmic enzymes in Prokarya [33], led Harding et al. [43] to speculate that the synthesis of ectoines might occur in these organelles of the protists.

Although the overall evolutionary relevance of horizontal gene transfer events from Pro- to Eukarya is still intensively debated [48], it is nevertheless well established that Eukarya can acquire novel metabolic traits and stress resistance determinants by stealing preformed gene clusters from either Bacteria or Archaea [46,50,51]. It is likely that the acquisition and salt stress–responsive transcription of ectoine/5-hydroxyectoine biosynthetic genes from its food prey will provide a distinct advantage to *H*. *seosinensis* in its high-salinity habitat [43] because ectoines are very potent osmostress protectants [33,34,36]. The occurrence of ectoine/5-hydroxyectoine biosynthetic genes in the eukaryote *H*. *seosinensis* does not seem to represent an isolated incident. In extended database searches of eukaryotic genomes, Harding et al. [43] discovered *ectA*- and *ectC*-related sequences in previously reported transcriptional profiles of at least six other protists and in the genome sequences of a considerable number of other Eukarya as well. This includes even the deuterostome animals *Branchiostoma floridae* and *Saccoglossus kowalevskii*. Interestingly, in a number of cases, fusions of the *ectA* and *ectC* open reading frames have occurred. In the dinoflagellate *Azadinium spinosum*, this fused open reading frame also contains *ectD*, suggesting the production of a potentially trifunctional ectoine/5-hydroxyectoine biosynthetic enzyme [43]. In all these cases, experimental verification of ectoine/5-hydroxyectoine production by the corresponding eukaryotic cells is eagerly awaited.

The phylogenetic relationship and genomic arrangement of the *H*. *seosinensis ectABC–ask_ect* gene cluster not only prompted Harding et al. [43] to advocate that these genes were acquired via lateral gene transfer from Prokarya. Their sporadic presence (or at least remnants of them) in various protists also suggested to these authors that the *ect* biosynthetic genes have spread horizontally between various halophilic unicellular eukaryotes [43]. These authors also recorded a number of cases in which salt-regulated genes unrelated to the synthesis of ectoines were part of gene duplication events, indicating that this process, along with several other lateral gene transfer events that they detected, was involved in creating genetic novelties [44], thereby aiding the adaptation of the bacteriovour *H*. *seosinensis* to its permanently high-saline habitat [41,52].

While the data provided by Harding et al. [43] are very suggestive of ectoine/5-hydroxyectoine biosynthesis in a selected group of Eukarya, definitive proof that this is actually the case in *H*. *seosinensis* was missing because these authors did not investigate the production of these solutes directly. The study by Weinisch et al., now published in *PLoS Biology* [45], convincingly fills this gap of knowledge by demonstrating the salt-dependent synthesis of ectoine by the halophilic heterotrophic ciliate *S*. *salinarum*. Although no genome sequence or transcriptional profile is currently available for *S*. *salinarum* that would allow the verification of the presence of the *ectABC* genes, data derived from ^1^H-NMR-spectroscopy provided unequivocal evidence for the synthesis of ectoine [45]. Interestingly, Weinisch et al. [45] also found through tracer experiments with externally provided radiolabeled choline that *S*. *salinarum* can synthesize glycine betaine through oxidation of this precursor. Even more surprising than the data on the de novo synthesis of ectoine was the finding by Weinisch et al. that externally provided ectoine, and to a lesser extent glycine betaine, enhanced growth under high-salinity conditions [45]. These observations imply that this protist, like many microorganisms [5,38], possesses transporter(s) for these compatible solutes, thereby allowing osmostress protection [45]. We note in this context that many microbial ectoine/5-hydroxyectoine producers live in high-saline ecosystems [33,34,36], and the active export or release of ectoines from these primary producers will make a major contribution of occurrence of ectoines in these habitats.

Taken together, *S*. *salinarum* employs a set of different imported or newly synthesized compatible solutes to cope with sustained osmotic stress, an adjustment strategy that is frequently found in Bacteria and Archaea as well (Fig 1) [5–7]. This concept is further buttressed by the finding of Harding et al. [43] that the expression of two predicted transporters for myo-inositol (Fig 2), a well-known compatible solute in Eukarya [8], were up-regulated in response to salt stress in *H*. *seosinensis*.

Finally, ectoines are not only effective osmostress protectants but they are also valuable nutrients for microorganisms [24]. The physiology and genetics of the catabolism of these nitrogen-rich compounds (Fig 2) have been intensively studied in the marine bacterium *Ruegeria pomeroyi* [53]. A unique ectoine-derived metabolite (N-α-L-acetyl-2,4-diaminobutyric acid), and not ectoine or hydroxyectoine themselves, serves as the inducer for the GabR/MocR-type regulatory protein EnuR that controls the transcription of the ectoine/5-hydroxyectoine import and catabolic gene clusters present in many microorganisms [53]. Interestingly, the cocultivation of *R*. *pomeroyi* with the diatom *Thalassiosira pseudonana* induces the transcription of this operon [54], implying that the diatom produces ectoine (and perhaps also 5-hydroxyectoine) and releases these osmolytes into the marine environment from which *R*. *pomeroyi* can then scavenge them as nutrients [53].

Taken together, the recently reported data by Harding et al. [43] and Weinisch et al. [45] on ectoine/5-hydroxyectoine biosynthesis by halophilic protists as osmostress protectants, and the report by Landa et al. [54] on the remodeling on the transcriptional profile of the ectoine/5-hydroxyectoine consumer *R*. *pomeroyi* through metabolites released by the diatom *T*. *pseudonana*, break new ground. They highlight the unexpected ecophysiological importance of ectoines as stress protectants in a selected group of halophilic Eukarya. These studies also underscore the role of ectoines as mediators of ecophysiological interactions between Pro- and Eukarya in salt-stressed ecosystems beyond a level that was assumed until recently [33,34,36,53].

## Abbreviations

DMS: dimethylsulfide
DMSP: dimethlysulfoniopropionate
MscL: mechanosensitive channel large
MscM: mechanosensitive channel mini
MscS: mechanosensitive channel small
SAM: *S*-adenosylmethionine
